# Targeting the MAPK signaling pathway: implications and prospects of flavonoids in 3P medicine as modulators of cancer cell plasticity and therapeutic resistance in breast cancer patients

**DOI:** 10.1007/s13167-025-00407-6

**Published:** 2025-04-10

**Authors:** Peter Kubatka, Bianka Bojkova, Natalia Nosalova, Mykhailo Huniadi, Samson Mathews Samuel, Bini Sreenesh, Gabriela Hrklova, Karol Kajo, Slavomir Hornak, Dasa Cizkova, Rostyslav Bubnov, Ivica Smokovski, Dietrich Büsselberg, Olga Golubnitschaja

**Affiliations:** 1 Centre of Experimental and Clinical Regenerative Medicine, Small Animal Clinic, University of Veterinary Medicine and Pharmacy, 041 81 Kosice, Slovakia; 2 Department of Biology and Ecology, Pedagogical Faculty, Catholic University in Ružomberok, 034 01 Ružomberok, Slovakia; 3 Department of Animal Physiology, Institute of Biology and Ecology, Faculty of Science, Pavol Jozef Šafárik University in Košice, Košice, 040 01 Slovakia; 4 Department of Physiology and Biophysics, Weill Cornell Medicine in Qatar, Education City, 24144 Doha, Qatar; 5 Department of Pathology, St. Elisabeth Oncology Institute, 812 50 Bratislava, Slovakia; 6 Small Animal Clinic, University of Veterinary Medicine and Pharmacy, 041 81 Kosice, Slovakia; 7 Institute of Neuroimmunology, Slovak Academy of Sciences, 845 10 Bratislava, Slovakia; 8 Clinical Hospital “Pheophania”, Kyiv, Ukraine; 9 Zabolotny Institute of Microbiology and Virology, National Academy of Sciences of Ukraine, Kyiv, Ukraine; 10 University Clinic of Endocrinology, Diabetes and Metabolic Disorders, Skopje, North Macedonia; 11 Faculty of Medical Sciences, University Goce Delcev, Stip, North Macedonia; 12 Predictive, Preventive and Personalised (3P) Medicine, Department of Radiation Oncology, University Hospital Bonn, Rheinische Friedrich-Wilhelms-Universität Bonn, 53127 Bonn, Germany

**Keywords:** Breast carcinoma, Predictive preventive personalized medicine (PPPM / 3PM), Cancer chemo-resistance, Cell plasticity, Anti-cancer therapy, Re-sensitization, Flavonoids, MAPK signaling, Multi-level diagnostics, Artificial intelligence, Big data interpretation, Patient phenotyping and stratification, Treatments tailored to individualized patient profile, Primary and secondary care, Improved individual outcomes, Health policy

## Abstract

Cancer drug resistance poses a significant challenge in oncology, primarily driven by cancer cell plasticity, which promotes tumor initiation, progression, metastasis, and therapeutic evasion in many different cancers. Breast cancers (BCs) are a prominent example of that, with an estimated 2.3 million new cases and 670,000 BC-related deaths registered worldwide annually. Triple-negative BC is especially challenging for treatments demonstrating particularly aggressive disease course, an early manifestation of metastatic disease, frequent drug-resistant cancer types, and poor individual outcomes. Although chemosensitizing agents have been developed, their clinical utility in oncology remains unproven. The mitogen-activated protein kinase (MAPK) pathway is considered a critical regulator of intracellular and extracellular signaling highly relevant for both — genetic and epigenetic modifications. Dysregulation of the MAPK signaling pathways plays a significant role in conferring chemoresistance in BC. Contextually, targeting the MAPK pathway represents a promising strategy for overcoming drug resistance and enhancing the therapeutic efficacy of anticancer agents in BC treatment. On the other hand, flavonoids, a prominent class of phytochemicals, are key modulators of MAPK signaling. Flavonoids interact with the ERK, JNK, p38, and ERK5 pathways of the MAPK signaling cascade and present a promising avenue for developing novel anti-cancer therapies and re-sensitizing agents for the treatment of BC. Compounds such as quercetin, kaempferol, genistein, luteolin, myricetin, EGCG, baicalein, baicalin, nobiletin, morin, delphinidin, acacetin, isorhamnetin, apigenin, silymarin, among others, have been identified as specific modulators of MAPK signaling, exerting complex downstream effects in BC cells increasing therewith drug efficacy and suppressing tumor growth and aggressivity. These properties reflect mechanisms of great clinical relevance to overcome therapeutic resistance in overall BC management. This article highlights corresponding mechanisms and provides clinically relevant illustrations in the framework of 3P medicine for primary (protection of individuals at high risk against health-to-disease transition) and secondary care (protection against metastatic BC progression). 3PM novelty makes good use of patient phenotyping and stratification, predictive multi-level diagnostics, and application of Artificial Intelligence (AI) tools to the individualized interpretation of big data — all proposed for cost-effective treatments tailored to individualized patient profiles with clear benefits to patients and advanced BC management.

## Introduction

### Disruption of intracellular signaling is associated with carcinogenesis

Cellular signaling pathways are organized as modular networks with constant intercommunication. The signaling components within these pathways exhibit binary, switch-like interactions, wherein the binding of two proteins results in either direct or indirect activation or inhibition of downstream molecular targets in the signaling cascade. Pathological alterations in these signaling pathways are maintained through genetic, transcriptomic, and epigenetic changes, impacting various mechanisms, including cell fate determination [1]. Disruption of intracellular signaling can impact crucial processes associated with carcinogenesis, including resistance to apoptosis, uncontrolled cell proliferation, angiogenesis, invasion, metastasis, survival and plasticity of cancer stem cells, and resistance to chemotherapy [2]. Mitogen-activated protein kinases (MAPKs) are crucial in human carcinogenesis, influencing tumor progression based on the mutational landscape and cellular context. Depending on alterations in their downstream effectors, MAPK signaling can either drive oncogenic pathways or exert tumor-suppressive functions [3, 4].

MAPKs constitute a family of serine/threonine protein kinases that mediate various cellular responses to diverse extracellular and intracellular stimuli, including mitogens, osmotic stress, heat shock, and proinflammatory cytokines. These kinases play a pivotal role in regulating cellular processes such as proliferation, gene expression, differentiation, mitosis, survival, and apoptosis [5]. The most extensively studied MAPKs belong to the conventional subfamilies, which include extracellular signal-regulated kinases 1 and 2 (ERK1/2), c-Jun N-terminal kinases (JNK1–3), p38 isoforms (α, β, γ, and δ), and the ERK5 pathway. In addition to these, atypical MAPKs—such as ERK3/4, ERK7/8, and Nemo-like kinase (NLK)—exhibit distinct regulatory mechanisms and specialized biological functions [5].

### MAPK signaling associated modulation of therapeutic resistance in breast cancer

According to the World Health Organization (WHO), in 2022, approximately 2.3 million women were diagnosed with breast cancer (BC), resulting in 670,000 deaths worldwide [6]. Acquired drug resistance remains a key challenge in clinical oncology and is the primary contributor to cancer-related deaths, although substantial advancements have been made in cancer therapies. This resistance emerges following initial anti-cancer treatment and leads to a progressive decline in therapeutic effectiveness. This process is driven by multiple mechanisms, including the upregulation of key oncogenic driver genes, the accumulation of mutations leading to dysregulated expression of molecular targets, and modifications within the tumor microenvironment (TME) [7]. Studies have explored various aspects of MAPK signaling relevant to drug resistance, including the ERK1/2, JNK, p38 MAPK, and ERK5 pathways and epigenetic and metabolic alterations associated with MAPK signaling [4, 8, 9]. Extensive clinical evidence documents that widespread phosphorylation and activation of MAPK inhibit tumor cell death and promote resistance to various standard chemotherapeutic agents [1, 3]. This mechanism is, therefore, linked to a poorer prognosis for tumor recovery. This process is further related to poor prognostic outcomes in tumor recovery. Importantly, clinical investigation indicates that commonly used chemotherapy agents in BC, including taxanes, anthracyclines, and platinum-based drugs, frequently activate the MAPK signaling pathway [10–13].

### Mechanisms of anticancer therapy resistance associated with MAPK signaling

Based on the above-mentioned findings, the MAPK pathway has emerged as a promising target for developing novel anticancer therapies for BC. Numerous chemosensitizing agents have been investigated in clinical research to overcome BC resistance. However, their therapeutic application has been largely limited due to significant adverse effects in cancer patients or the rapid recurrence of drug resistance [14, 15]. Through cell plasticity, tumor cells can reversibly shift between proliferative, metastatic phenotypes and dormant, drug-tolerant states, thus undermining the effectiveness of targeted therapies. This broad plasticity enables tumor cells to adapt through various yet functionally similar mechanisms, such as epithelial-mesenchymal transition (EMT), trans-differentiation, and the acquisition of cancer stem cell (CSC) traits [16–20].

### Flavonoids as potential modulators of cancer cell plasticity

With the advancement of technologies for isolating and identifying natural compounds, the potential of these products to address cancer multidrug resistance has gained significant attention. Natural compounds, owing to their ability to target multiple biological systems, provide a versatile approach to overcoming drug resistance through various mechanisms, including the modulation of cell plasticity [21–23]. Flavonoids, a diverse class of over 8000 known compounds widely distributed in plants, foods, and herbs, are prominent examples of such multi-target agents. These compounds exhibit numerous beneficial effects, including antioxidant, immunomodulatory, anti-inflammatory, and anticancer activities [24–26]. In addition to these effects, flavonoids can modulate cancer cell adaptability, influencing cellular plasticity and aiding in the reversal of tumor resistance to chemotherapy with minimal toxicity [7, 27]. While the antioxidant properties of flavonoids have been extensively studied, emerging evidence indicates that flavonoids and their metabolites in vivo exhibit functions beyond conventional antioxidant activity. Instead, they may influence cellular systems by directly modulating a range of signaling pathways. These signaling pathways include phosphoinositide 3-kinase (PI3K), Akt/protein kinase B (PKB), tyrosine kinases, protein kinase C (PKC), and the MAPK cascade [28]. Various flavonoids have been found to interact with the ERK, JNK, and p38 pathways within the MAPK signaling cascade [29]. These findings support the growing interest in exploring flavonoids as potential agents for novel anticancer therapies, specifically for BC, or as effective chemo-preventive agents by targeting cellular plasticity and adaptive mechanisms of resistance.

### Aim of the study

The anticancer properties of flavonoids, extensively demonstrated in preclinical studies, hold significant clinical potential, particularly when integrated into personalized anti-cancer treatment strategies tailored to individual patient profiles. To the best of our knowledge, no current publication provides a comprehensive summary of the role of flavonoids in modulating cellular plasticity in BC via the MAPK signaling pathway. This study aims to bridge this knowledge gap by investigating the capacity of flavonoids to regulate cancer cell plasticity, thereby improving the responsiveness of BC cells to conventional therapies through the modulation of MAPK-related signaling pathways. The strategic development of combination anti-cancer therapies incorporating new molecules, including natural substances (e.g., flavonoids), is essential to enhance clinical outcomes for oncology patients [7, 30].

### Source of the analyzed research data, inclusion, and exclusion criteria

Data were obtained from the PubMed database through the use of relevant keywords and Medical Subject Headings (MeSH) terms. The search included terms such as “breast carcinoma,” “cell plasticity,” “resistance,” “MAPK signaling,” “flavonoids,” and various flavonoid subclasses, including “flavanones,” “flavonols,” “flavones,” “flavanols,” “isoflavonoids,” “chalcones,” and “anthocyanidins.” Additional terms such as “radiotherapy,” “chemotherapy,” “targeted therapy,” “3 PM,” and other related concepts were also incorporated into the search strategy.

The inclusion criteria for Heading 3 were defined as follows: (1) studies investigating the effects of flavonoids in BC; (2) experimental groups treated with nanomaterials in combination with flavonoids; (3) studies linking flavonoid effects to the modulation of MAPK signaling; (4) research conducted in vitro, in vivo using animal models or human subjects with the application of natural or synthetic flavonoids; (5) controlled experimental designs; (6) studies involving pure flavonoids or combination therapies; and (7) research highlighting the influence of flavonoids on cancer cell plasticity and/or the reversal of chemoresistance through modulation of MAPK signaling.

The exclusion criteria for Heading 3 were defined as follows: (1) non-original full research articles; (2) interventions involving phytochemicals other than flavonoids or studies lacking clear information on the dose and duration of flavonoid administration; (3) studies examining the application of flavonoids in treating cancers beyond BC or their effect on signaling pathways other than MAPK.

## Reciprocity between modulation of cellular plasticity and the emergence of chemotherapeutic resistance in breast cancer mediated by MAPK signaling

### The role of MAPK signaling in carcinogenesis

**Fig. 1:**
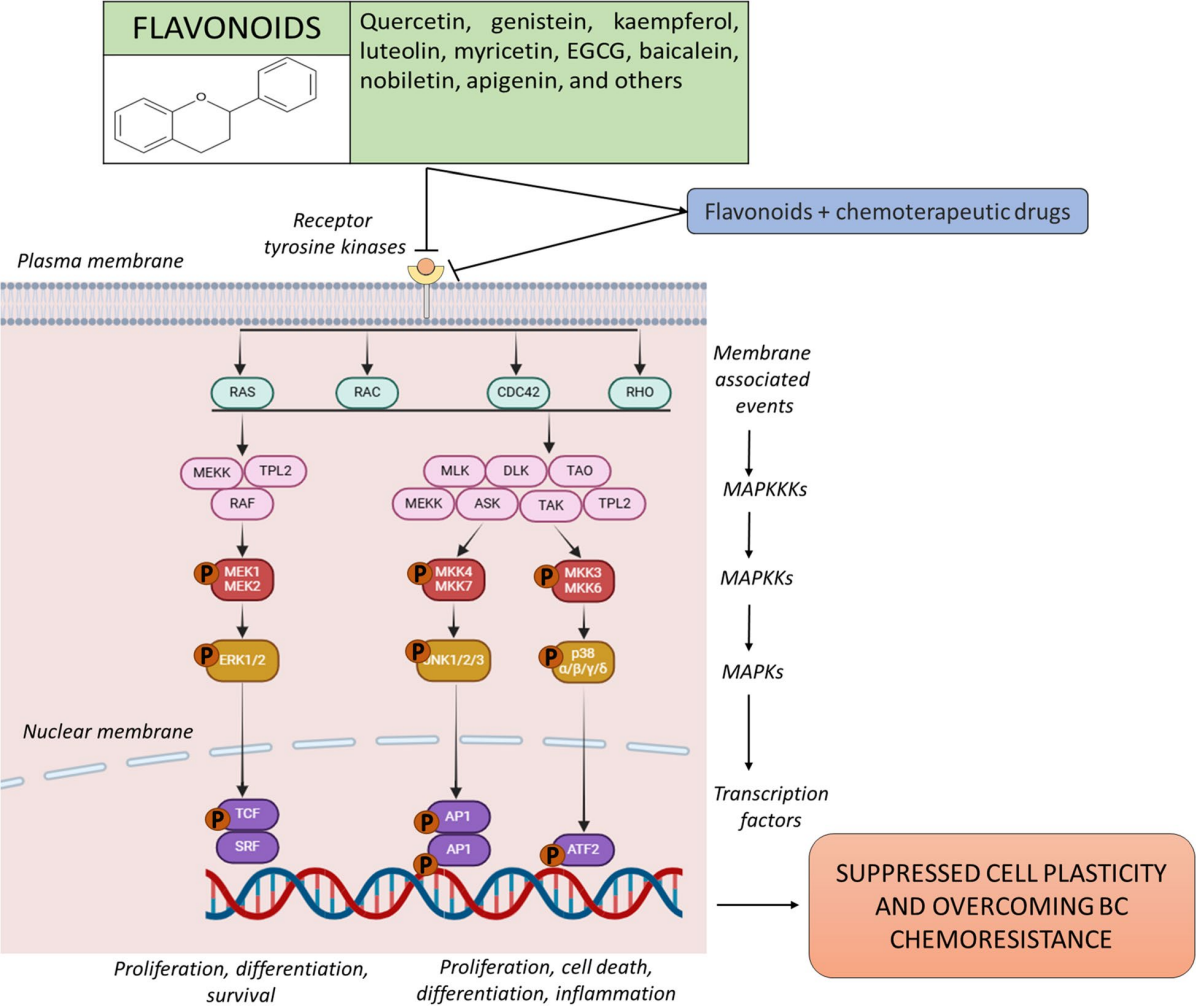
The membrane receptor tyrosine kinases-linked anti-BC effects of flavonoids as modulators of the MAPK signaling pathway, highlighting their capacity to function as sensitizers in chemoresistant BC and/or as chemopreventive agents. The MAPK cascade consists of three hierarchical levels: MAPK kinase-kinase (MAPKKK), MAPK kinase (MAPKK), and MAPK. This signaling axis integrates extracellular stimuli, primarily via Gprotein-coupled receptors, to elicit diverse cellular responses. Within the canonical MAPK/ERK pathway, MAPKKKs comprise A-RAF, B-RAF, and RAF-1 (C-RAF) kinases, which activate the MAPKKs, MEK1, and MEK2. These, in turn, phosphorylate and activate the terminal effectors, ERK1 and ERK2, thereby propagating downstream signaling. Additionally, membrane-associated molecular interactions involve the modulation of small GTPases such as Rac, CDC42, and Rho, which serve as upstream regulators of the MAPK-JNK and MAPK-p38 signaling branches, further contributing to the pathway's complexity

MAPKs are serine/threonine kinases that facilitate the transduction of extracellular signals into various cellular responses. As one of the most evolutionarily conserved signaling cascades, MAPKs play an essential role in a wide range of physiological processes. In eukaryotic cells, multiple MAPK pathways work in concert to govern crucial cellular functions, such as gene expression, cell cycle progression, metabolism, migration, survival, apoptosis, and differentiation [5, 31]. The MAPK signaling network consists of four distinct pathways, each formed by a specific signaling family: the classical MAPK/ERK, the c-Jun N-terminal kinase (JNK), the p38, and the ERK5 (or Big MAP kinase-1 (BMK-1) pathways [3]. These kinases are organized in a hierarchical cascade from upstream to downstream, progressively moving closer to the nucleus. The levels are MAPK kinase-kinase (MAPKKK), MAPK kinase (MAPKK), and MAPK. The kinases MAPKKK, MAPKK, and MAPK constitute a signaling cascade that integrates G-protein-derived signals, leading to the activation of diverse biological responses. In the canonical MAPK/ERK pathway, the MAPKKK includes three types: A-RAF, B-RAF, and RAF-1 (also known as C-RAF) kinases. Directly downstream are the MAPKKs, consisting of MEK1 and MEK2. At the final level are ERK1 and ERK2, which serve as the terminal effectors of the MAPK pathway (Fig. 1) [3].

The MAPK signaling pathway is crucial in the initiation and progression of human cancer [32, 33]. In this context, extracellular signal-regulated kinases 1 and 2 (ERK1/2) are highly conserved serine/threonine kinases found in all eukaryotic cells, serving as critical modulators of cellular signaling in both normal and pathological states. ERK1/2 expression is essential for normal development, while their aberrant activation is a major driver of cancer initiation and progression. Among the MAPK signaling pathways, the Ras/Raf/MEK/ERK cascade is the most critical, playing an essential role in tumor cell survival, proliferation, and progression [32]. Under basal conditions, ERK is predominantly located in the cytoplasm; however, upon activation, it translocates to the nucleus, where it regulates transcription factors and controls gene expression [34].

Cancer is a highly heterogeneous disease characterized by diverse cellular states and phenotypes. This variability, termed cancer cell plasticity, enables malignant cells to dynamically adapt to environmental changes, fostering tumor heterogeneity and therapeutic resistance. Key mechanisms underlying this adaptability include epithelial-mesenchymal transition and the acquisition of stem cell-like traits, both of which drive tumor progression and complicate treatment strategies. Although targeted therapies have advanced considerably, the ability of cancer cells to evolve and develop resistance continues to hinder long-term treatment efficacy [35]. Several examples demonstrate the link between MAPK signaling and the regulation of cancer cell plasticity. In this regard, current evidence indicates that the interaction between the TGF-β-induced Smad pathway and the MAPK signaling is crucial in determining the ultimate cellular response to TGF-β, particularly in the context of EMT [17, 18]. Another study identified a key role for p38γ MAPK in EMT and CSCs while uncovering a novel signaling through which p38γ MAPK promotes tumorigenesis [19]. Another mechanism included in the modulation of cell plasticity via the MAPK is the activation of the Ras/Raf-1 signaling pathway, which leads to the phosphorylation of MAPK and MEK in carcinoid tumor cells, resulting in their morphological trans-differentiation [20].

### MAPK and resistant breast carcinoma

BC ranks among the most common cancer diagnoses and stands as the primary cause of cancer-related mortality in women globally [36]. In mammalian cells, four principal MAPK signaling pathways are implicated in breast disease: (a) the extracellular signal-regulated kinase (ERK)1/2 pathway, (b) the c-Jun N-terminal kinase (JNK) pathway, (c) the p38 pathway, and (d) the ERK5 pathway [37] (Fig. 1). Signaling through the ERK1/2 pathway has been recognized as a key contributor to various forms of human BC and in multiple experimental models of BC progression [32]. Growth factors and cytokines activate the ERK1/2 signaling cascade via receptor tyrosine kinases, G-protein-coupled receptors, and non-nuclear steroid hormone receptors. The substrates of ERK1/2 include transcriptional regulators, modulators of apoptosis, and steroid hormone receptors, such as the estrogen receptor (ER)α [38, 39]. Phosphorylation of ERK1/2 substrates leads to various biological effects, including promoting proliferation, epithelial-to-mesenchymal transition, survival, angiogenesis, motility, and invasiveness [37, 40].

While the molecular basis of BC resistance to standard chemotherapy remains incompletely elucidated, several potential mechanisms have been identified. Resistance mechanisms in BC include increased drug efflux, enhanced DNA repair, senescence escape, epigenetic alterations, tumor heterogeneity, TME, and EMT [41]. The activation of MAPK kinase plays a key role in several of these processes, particularly in EMT, cell proliferation, and adaptation to the TME [4, 41–43]. Eralp et al. [13] concluded that MAPK expression may play a significant role in the emergence of chemoresistance, potentially defining a distinct molecular subgroup within triple-negative breast cancer (TNBC). Moreover, their findings suggest that tumor proliferation in this subgroup may be governed by different molecular mechanisms before and after initial recurrence, likely promoting the selection of more aggressive clones as the disease progresses and metastasizes. Additionally, a separate clinical study identified a correlation between MAPK signaling and epidermal growth factor receptor (EGFR) expression in TNBC tissues. Patients with high MAPK and EGFR expression were more likely to have lymph node metastasis, advanced disease stages, tumor recurrence, and distant metastases. These patients also had shorter overall survival compared to those with lower expression levels [44]. A preclinical in vitro study demonstrated that TGF-β1, a crucial cytokine secreted by chemoresistant BC cells, promotes chemoresistance in cancer-associated fibroblasts (CAFs) by activating the p44/42 MAPK signaling pathway. Genetic and pharmacological inhibition of TGF-β1 suppresses p44/42 MAPK activation, restoring chemosensitivity in CAFs [45].

Several studies have shown that MAPK proteins, including ERK and p38, regulate MDR1 expression, thereby contributing to taxane resistance in BC [3, 46, 47]. Besides regulation via STAT3, MDR1 expression in taxane-resistant BC is also transcriptionally controlled by EGR1, which ERK1/2 regulates [48]. Furthermore, elevated MAPK expression has been associated with anthracycline resistance and an increased risk of recurrence in patients with TNBC [13]. Platinum-based chemotherapeutic agents, among the most potent anticancer drugs, are widely employed in the treatment of various solid tumors, including BC. However, resistance to these agents has been linked to MAPK signaling, prompting ongoing clinical trials investigating small-molecule inhibitors targeting the MAPK cascade as a potential strategy to overcome platinum drug resistance [49, 50].

The phosphorylation of ERK1/2, JNK, p38, and ERK5 substrates plays a crucial role in regulating key biological processes that contribute to cancer cell plasticity and therapeutic resistance. These signaling pathways drive cellular proliferation, EMT, and survival, facilitating tumor progression and adaptation to environmental stressors. Additionally, they promote angiogenesis, motility, and invasiveness, enhancing metastatic potential and complicating treatment efficacy. By enabling dynamic phenotypic changes, dysregulation of these pathways contributes to decreased sensitivity of anticancer drugs, underscoring their significance as potential therapeutic targets in cancer treatment [7, 37, 40].

The role of the above-mentioned signaling pathways in the modulation of tumor plasticity is described in the following subchapters.

### The involvement of the ERK1/2 signaling pathway in the development of resistance to anticancer chemotherapy

ERK1/2 is a key downstream mediator in the RTK/RAS/BRAF/MEK signaling cascade, serving as a critical intersection point for various cellular pathways that modulate apoptosis, cell cycle, chemoresistance, immune resistance, and immune evasion. ERKs function as essential signaling hubs, precisely detecting changes in the TME and orchestrating adaptive responses in cancer cells [51]. Approximately 40% of human cancers exhibit alterations in the RAS-RAF-MEK-ERK signaling cascade, primarily driven by mutations in the upstream regulator RAS (around 30%) and the downstream effector BRAF (approximately 10%) [52]. Due to its role as a mediator of multiple extracellular signals and its involvement in several receptor tyrosine kinase (RTK) pathways, ERK may be considered a potential vulnerability in cancer chemotherapy [53]. In tumor cells, ERK1/2 inhibition typically counteracts chemoresistance and alleviates tumor-induced immunosuppression. However, targeting ERK1/2 in tumor-infiltrating cells poses significant risks, as such inhibitors may impede the proliferation of immune-effector cells and immune-suppressive cells.

The clinical efficacy of ERK1/2 inhibitors has fallen short of expectations due to various limitations. One major factor is the nature of oncogenic activation; for instance, sustained, continuous stimulation, as opposed to transient, pulsatile activation, significantly influences the effectiveness of ERK inhibition [54]. Secondly, similar to MEK and BRAF inhibitors, resistance to ERK1/2 inhibitors has been reported, driven by mutations or amplification of ERK proteins [55]. Nonetheless, resistance can be mitigated through combination therapies targeting multiple components of the ERK1/2 signaling pathway. Thirdly, ERK1/2 regulates both the mitosis and differentiation of non-transformed cells, meaning its suppression could potentially disrupt normal tissue physiology, leading to severe adverse effects [56].

### The involvement of the JNK pathway in mediating drug resistance

The c-JUN N-terminal kinase (JNK) pathway is crucial for controlling cellular proliferation and survival [57, 58]. JNK regulates immune responses, endothelial cells, CSCs, and stromal cells [59]. Inhibiting the JNK pathway may enhance therapeutic efficacy or hinder tumor progression within the TME. The JNK family comprises three isoforms encoded by distinct genes: JNK1, JNK2, and JNK3. While JNK1 and JNK2 are ubiquitously expressed across tissues, JNK3 exhibits tissue-specific expression.

JNK has been identified as a kinase that conveys oncogenic RAS-driven signaling to the nucleus by phosphorylating and activating JUN transcription factors, thereby regulating gene expression [60]. Its role in cancer is dual-faceted, functioning as both a tumor promoter and suppressor based on its influence on apoptosis and autophagy pathways. Additionally, JNK plays complex roles within the heterogeneous TME, contributing to various tumor-promoting processes in a context-dependent manner, influenced by cancer type and disease stage. The JNK pathway modulates multiple stress responses and chronic inflammatory conditions, as well as influences diverse cell populations within the TME [61]. Dysregulated activation of this pathway is observed in various cancers, where it mainly supports tumor progression. Furthermore, activation of the JNK pathway has been linked to developing resistance to anticancer therapies [61, 62]. Comprehensive studies on isoform-specific JNK-mediated events in TME development and strategies for identifying patients likely to respond are essential to leveraging JNK modulation as a potential novel therapeutic approach in clinical cancer cures.

### The contribution of the p38 signaling pathway to drug resistance

The regulation of the p38 signaling pathway is intricate and multifaceted. While its activation typically occurs through two upstream MAPKKs, MKK3 and MKK6, alternative mechanisms also contribute. These mechanisms involve activation through the interaction with TA1 (TAK1 binding protein 1) and phosphorylation by the tyrosine kinases ZAP70 and LCK, both of which promote p38 autophosphorylation at critical regulatory residues [63]. Furthermore, p38 exists in four isoforms—p38α, p38β, p38γ, and p38δ—encoded by distinct genes [64].

The p38 MAPK signaling pathway is widely recognized for mediating environmental stress signals. While numerous components and regulatory mechanisms within this cascade have been partially characterized, the pathway is implicated in a diverse array of cellular processes, many of which remain insufficiently understood at the molecular level [65]. Initially identified as a tumor-suppressor kinase due to its ability to inhibit RAS-driven transformation, p38 has also been shown, through extensive experimental evidence, to act as a tumor promoter under certain conditions [66]. The activity of p38 kinase may promote cancer cell proliferation, survival, migration, or resistance to stress and chemotherapeutics, offering a preclinical and clinical basis for investigating p38 kinase inhibitors as a potential therapeutic strategy for cancer, including BC. The p38 MAPK signaling pathway operates downstream of several pathways specific to CSCs, where it plays a crucial role in CSC formation, self-renewal, and maintenance and also significantly contributes to metastatic progression [67–69].

### Impact of ERK5 on the cancer resistance

Like other members of the MAPK family, ERK5 protein kinase plays a critical role in fundamental cellular processes such as proliferation, differentiation, migration, and survival. While the oncogenic functions of conventional MAPK pathways are well-documented, the MEK5-ERK5 signaling axis has only recently emerged as a focus of cancer research [70]. The MEK5-ERK5 signaling cascade has recently been recognized as a critical driver of tumorigenesis and metastatic progression. In this regard, elevated expression of MEK5 in BC tissue compared to normal counterparts underscores its potential involvement in tumor progression [71]. Montero et al. identified ERK5 expression in most early-stage BC patients, with overexpression observed in 20% of cases [72]. Notably, ERK5 overexpression has been linked to decreased disease-free survival and is prevalent in the more aggressive TNBC subtype [72, 73]. In BC cell lines overexpressing HER2, ERK5 remains constitutively active, and its inhibition significantly reduces the proliferation of HER2 + cells [74]. Moreover, emerging evidence suggests that the MEK5-ERK5 pathway is pivotal in mediating drug resistance in cancer therapy [75]. The study identified ERK5 as a key mediator of resistance to BRAFi/MEKi and ERK1/2i therapies [76]. The MAPK signaling cascade exhibits significant redundancy and overlaps in the downstream targets of the MEK1/2 and MEK5 pathways [5]. Consequently, additional research is required to investigate the potential synergistic outcomes of the concurrent inhibition of MEK1/2 and MEK5. This dual-targeting strategy may represent a promising avenue for treating aggressive cancers by delaying the onset of drug resistance and improving therapeutic efficacy in patients. Building on this knowledge, future efforts should focus on identifying and developing selective, clinically viable MEK5–ERK5 inhibitors [71].

## The regulatory role of flavonoids in MAPK signaling and its impact on cancer cell plasticity

The MAPK pathway promotes chemoresistance, survival, and metastasis in aggressive cancers, offering the potential for new therapies [77]. Several inhibitors targeting the MAPK signaling pathway have been employed in clinical treatments. However, their therapeutic outcomes remain suboptimal due to challenges such as drug resistance, genomic instability, and significant adverse effects [78]. In contrast, natural products present distinct advantages, including minimal side effects, potent therapeutic efficacy, broad availability, and diverse biological activities adaptable to individual variations. Notably, their capacity to target multiple molecular pathways positions them as a promising strategy for disease management [29]. Flavonoids represent a vital class of phytochemicals that regulate MAPK signaling and interfere with key cellular processes driving acquired cancer cell plasticity and therapeutic resistance [79, 80]. Research, including findings from our laboratory and other studies, demonstrates that flavonoids enhance tumor cell responsiveness to chemotherapy and radiotherapy, especially in the context of multidrug resistance and tumor relapse [27, 81, 82]. Moreover, flavonoids mitigate treatment-associated toxicity and safeguard healthy cells from cytotoxic damage. Their synergistic interaction with anticancer therapies promotes apoptosis, suppresses angiogenesis, inhibits tumor invasion and metastasis, and modulates resistance pathways, thereby enhancing overall therapeutic efficacy while minimizing systemic toxicity [7, 83, 84]. These multifaceted properties highlight the potential of flavonoids as modulators of cancer cell plasticity and resistance, primarily through the regulation of MAPK signaling pathways, including ERK1/2, JNK, p38, and ERK5.

### Flavonoids and the ERK1/2 signaling pathway

Chen et al. reported that quercetin attenuates the aggressive phenotype of TNBC by inhibiting the EMT pathway mediated by IGF1/IGF1R signaling. Their study demonstrated that quercetin suppresses IGF1R activation and its downstream kinases, Akt and ERK1/2, in a dose-dependent manner within the MDA-MB-231 cell line [85]. In another study, quercetin effectively suppressed epinephrine-induced cell cycle progression and migration of TNBC cells by targeting the β2-adrenergic receptor (β2-AR)/ERK1/2 signaling pathway [86]. The combined administration of doxorubicin and cyclophosphamide (AC) is a standard chemotherapy regimen for TNBC; however, its clinical application is limited by severe cardiotoxicity in cardiomyocytes. In vitro studies revealed that quercetin mitigates AC-induced cardiotoxicity by reducing reactive oxygen species (ROS) accumulation and activating the ERK1/2 pathway in cardiomyocytes. Importantly, quercetin enhances the antitumor efficacy of AC in TNBC cells by reducing ROS accumulation and inhibiting the ERK1/2 signaling pathway [87]. Quercetin and Epigallocatechin gallate (EGCG) have also shown protective activities against leptin-induced proliferation of MCF-7 cells by significantly attenuating leptin-triggered ERK1/2 phosphorylation [88]. Additionally, quercetin suppresses tumor invasion in MCF-7 cells by inhibiting matrix metalloproteinase-9 (MMP-9) activity by downregulating the PKCδ/ERK/AP-1 signaling pathway. [89]. These findings collectively highlight quercetin’s therapeutic potential in BC treatment via the modulation of the ERK1/2 pathway.

Bong-Woo et al. demonstrated that sustained activation of the ERK signaling pathway is crucial for kaempferol-induced apoptosis in MCF-7 cells, with this effect being notably enhanced under 3D culture conditions [90]. Kaempferol treatment markedly decreased the viability of MCF-7 cells while exerting minimal effects on the viability of MDA-MB-231 BC cells or breast epithelial HC-11 cells. Kaempferol induced sustained ERK activation, along with the activation of MEK1 and ELK1. This apoptosis was effectively blocked by PD98059, a MEK inhibitor, overexpression of a kinase-inactive ERK mutant, or ERK knockdown via siRNA [90]. Additionally, kaempferol exhibited anti-estrogenic effects by downregulating key components of IGF-1R signaling, including phosphorylated insulin receptor substrate-1 (pIRS-1), pAKT, phosphorylated MEK1/2 (pMEK1/2), and pERK1/2, all of which are upregulated in response to estrogen (E2) stimulation [91]. These findings highlight kaempferol’s potential as a therapeutic agent targeting ERK-dependent apoptosis and estrogen-driven signaling pathways in BC.

A study on luteolin demonstrated that this compound induces apoptosis in BC cells via both caspase-dependent and caspase-independent mechanisms, involving nuclear translocation of apoptosis-inducing factor (AIF) mediated by the upregulation of the ERK and p38 signaling pathways. Genetic knockdown or pharmacological inhibition of ERK and p38 significantly attenuated luteolin-induced apoptosis [92]. Additionally, luteolin exhibits antitumor activity against TNBC by inhibiting cell growth and EMT. These effects are mediated via the blocking of the Ras/Raf/MEK/ERK signaling pathway, regulated by miR-203 [93].

Additionally, myricetin was described to reduce the viability of human BC MCF-7 cells, at least in part, by promoting apoptosis through modulation of the PAK1/MEK/ERK/GSK3β/β-catenin/cyclin D1/PCNA/survivin/Bax-caspase-3 signaling pathway [94]. Research on TNBC cell lines MDA-MB-231 and MDA-MB-468 further indicated that myricetin induces apoptosis via H2O2 generation and enhanced phosphorylation of ERK1/2 and p38 proteins. These results underscore the therapeutic efficacy of flavonoids in modulating multiple molecular pathways involved in BC [95].

Osthole suppressed cellular proliferation and induced cell cycle arrest in BT-474 and MCF-7 cells through the regulation of key regulatory genes. Additionally, it disrupted mitochondrial membrane integrity, led to calcium dysregulation and endoplasmic reticulum stress, and promoted apoptosis via Bax activation. Osthole also regulated Akt, ERK1/2 phosphorylation, and JNK-mediated apoptosis in BC cells [96]. The flavonols chrysosplenol D and casticin demonstrated selective cytotoxicity against TNBC cell lines, including MDA-MB-231, CAL-51, and CAL-148, as well as estrogen receptor-positive MCF-7 cells [97]. Among these, MDA-MB-231 cells, characterized by high baseline ERK1/2 activity and low AKT signaling, showed heightened sensitivity to chrysosplenol D. Both flavonols also inhibited MDA-MB-231 cell growth in vivo. Mechanistically, chrysosplenol D and casticin disrupted mitochondrial membrane potential and induced programmed cell death. Chrysosplenol D specifically activated ERK1/2 signaling, increased cytosolic ROS, and triggered autophagy in MDA-MB-231 cell lines. Notably, the lysosomal dysfunction and associated cytotoxicity caused by chrysosplenol D could be mitigated by ERK1/2 inhibition [97].

Delphinidin, a member of the anthocyanin class of flavonoids, is a primary pigment found predominantly in the floral tissues of plants. This natural compound inhibits HER2 and ERK1/2 signaling, thereby suppressing the proliferation of HER2-overexpressing TNBC cells. Delphinidin significantly downregulates HER2 signaling by reducing the phosphorylation of HER2, Akt, and ERK1/2. The MAPK signaling pathway appears more strongly affected than the PI3K pathway, suggesting that delphinidin preferentially targets the HER2-MAPK axis over HER2-PI3K signaling. In HCC1806 TNBC cells, short-term exposure to delphinidin (50 μg/mL) effectively inhibits ERK1/2 phosphorylation without altering Akt phosphorylation, while phosphorylation of other MAPK proteins, such as p38 and JNK, remains unaffected. Similarly, in MDA-MB-468 TNBC cells, delphinidin treatment (50 μg/mL) reduces ERK1/2 phosphorylation without impacting Akt activity. These findings highlight delphinidin’s selective disruption of ERK1/2-driven downstream signaling [98].

Numerous studies have highlighted the therapeutic efficacy of flavonoids in modulating the ERK signaling pathway in BC. Syringin has been demonstrated to suppress the cell cycle and migration of TNBC cells while inducing apoptosis by regulating the PI3K/AKT/PTGS2 and EGFR/RAS/RAF/MEK/ERK pathways [99]. Morin induces cell death in MDA-MB-231 cells by causing sustained cell cycle arrest via ERK activation and FOXM1 inhibition, which leads to the overexpression of p21 [100]. Acacetin, a naturally derived flavonoid, exhibits potent anticancer effects in T-47D and MDA-MB-231 BC cell lines by inducing cell cycle arrest, generating ROS, and causing DNA damage that triggers RIP1-dependent necroptotic cell death. This activity is mediated by sustained ERK1/2 activation driven by ROS production. However, pre-treatment with N-acetyl cysteine (NAC) suppressed ROS formation, partially attenuating ERK1/2 activation and reducing acacetin-induced cell death. [101]. Interestingly, at low doses, acacetin promotes proliferation in MCF-7 cells by triggering the ERK1/2, PI3K/AKT, and cyclin signaling pathways [102]. Silymarin has been shown to regulate BC cell proliferation and apoptosis in vitro and in vivo through modulation of the MAPK signaling pathway. Mechanistically, silymarin upregulates the expression of pro-apoptotic proteins, including Bax, cleaved poly-ADP ribose polymerase (PARP), cleaved caspase-9, and phosphorylated JNK (p-JNK), while suppressing anti-apoptotic markers such as Bcl-2, phosphorylated p38 (p-p38), and ERK1/2 (p-ERK1/2) [103]. In MCF-7 cells, pre-treatment with lipopolysaccharide (LPS) activated the phosphorylation of NF-κB p65, NF-κB inhibitor (IκBα), and ERK1/2. Nonetheless, co-administration with puerarin counteracted these effects [104].

The flavonoid sophoraflavanone G significantly inhibits the phosphorylation of AKT (Ser473), p38, ERK1/2, and JNK in MDA-MB-231 cells [105]. Glabridin, an isoflavone derived from licorice root, suppresses angiogenesis, migration, and invasion in MDA-MB-231 cells by targeting the focal adhesion kinase (FAK)/Rho, AKT, and ERK1/2 signaling pathways [106]. Nobiletin induces G0/G1 cell cycle arrest by inhibiting ERK1/2 signaling, leading to upregulation of p21 and downregulation of cyclin D1 expressions in MCF-7, HER2-positive SK-BR-3, and triple-negative MDA-MB-468 BC cell lines [107]. Baicalein (10–15 µM) reduces adhesion, migration, and invasion in MCF-7 and SK-BR-3 BC cells by suppressing ERK1/2 and AKT signaling pathways, which are initiated through E2-induced GPR30-mediated EGFR activation [108, 109]. Similarly, fisetin has been described to inhibit TPA-induced cell invasion in MCF-7 cells by blocking PKCα/ROS/ERK1/2 and p38 MAPK signaling pathways, reducing NF-κB activity, and subsequently downregulating matrix metalloproteinase (MMP)−9 expression [110]. Genistein induces G2/M cell cycle arrest in MDA-MB-231 cells via concentration- and time-dependent ERK1/2 activation. This effect is blocked by the MEK1/2 inhibitor PD98059. Genistein increases Ras and Raf-1 protein levels independently of PD98059 but requires ERK1/2 activation to upregulate c-Jun and c-Fos [111]. Isorhamnetin has been shown to inhibit the phosphorylation of Akt, mTOR, MEK1, and ERK1/2 in MCF7 and MDA-MB-468 cell lines. Targeting the Akt/mTOR and MEK/ERK pathways, isorhamnetin promotes programmed cell death by upregulating Bax, Bcl-2, and cleaved caspase-3 expression [112].

These findings emphasize the potential of various flavonoids to regulate key signaling pathways, particularly those involving ERK1/2, in regulating cell plasticity for managing BC (Table 1).

**Table 1:** Summary of the effects of flavonoids on BC cells by modulating specifically MAPK-ERK1/2 upstream and downstream signaling pathways

Flavonoid	Study details	Affected process	Mechanism	Ref
Quercetin	MDA-MB.231 cells in vitro and xenograft mouse models	Blocking the aggressive phenotype of TNBC by suppressing IGF1/IGF1R-mediated EMT	↓ IGF1R ↓ Akt ↓ Erk1/2	[85]
Quercetin	MDA-MB-231 and MDA-MB-468 cells in vitro, and 4T1 xenograft mice	Suppression of the TNBC driven by chronic stress	↓ β_2_-AR/ERK1/2 pathway	[86]
Quercetin	MDA-MB-231 cells, 4T1 mouse model	Enhancing doxorubicin–cyclophosphamide effectiveness in TNBC	↓ ROS accumulation ↓ ERK1/2 pathway	[87]
Quercetin and EGCG	MCF-7 cells in vitro	Decreased proliferation	↓ ERK1/2 phosphorylation	[88]
Quercetin	MCF-7 cells in vitro	Inhibition of tumor invasion via MMP-9	↓ PKCdelta/ERK/AP-1-signaling cascade	[89]
Kaempferol	MCF-7 cells in vitro	Apoptosis of BC	↑ ERK, MEK1, and ELK1 expression,	[90]
Kaempferol	MCF-7 cells in vitro and in vivo	Suppression of estrogen- and triclosan-induced BC	↓ expression of pIRS-1, pAkt, pMEK1/2, and ERK1/2	[91]
Luteolin	MCF-7, MDA-MB-231 and SK-BR-3 cells in vitro	Induction of apoptotic cell death	↑ pERK and p38	[92]
Luteolin	MDA-MB-453 and MCF-7 cells in vitro	Decreased viability and accelerated apoptosis	↑ miR-203 ↓ Ras/Raf/MEK/ERK signaling	[93]
Myricetin	MCF-7 cells in vitro	Induction of apoptosis	↓ PAK1/MEK/ERK/GSK3β/β-catenin/cyclin D1/PCNA/survivin/Bax-caspase-3 signaling	[94]
Myricetin	MDA-MB-231 and MDA-MB-468 cells in vitro	Induction of apoptosis	↑ H_2_O_2_ ↑ pERK1/2 and p38	[95]
Osthole	BT-474 and MCF-7 cells in vitro	Disruption of MMP, triggering calcium imbalance and ER stress, and activation of apoptosis	↑JNK in both cell lines, ↓ Akt and ERK1/2 signaling in BT-474 cells ↑ Akt and ERK1/2 pathways in MCF-7 cells	[96]
Chrysosple- nol D and casticin	MDA-MB-231, CAL-51, CAL-148, as well as MCF7 cells in vitro	Disruption of MMP, lysosomal dysfunction, apoptosis, and autophagy induction	↑ ERK1/2 signaling ↑ ROS	[97]
Delphinidin	ER-positive, triple negative, and HER2-overexpressing BC cell lines in vitro, i.e. HCC1806, MDA231, MDA468, SKBR3, MDA453, BT474, and MCF7	Inhibition of proliferation, anchorage-independent growth, and induction of apoptosis	↓ MAPK signaling in TNBC ↓ HER2 ↓ Akt ↓ ERK1/2	[98]
Syringin	MDA-MB-231 and MCF-7 cells in vitro	Inhibition of proliferation and migration and promotion of apoptosis	↓ PI3K-AKT-PTGS2 and EGFR-RAS-RAF-MEK-ERK pathways	[99]
Morin	MDA-MB-231 cells in vitro	Cell death, cell cycle arrest	↑ ERK ↓ FOXM1 ↑ p21	[100]
Acacetin	T-47D and MDA-MB-231 cells in vitro	Cell cycle arrest	↑ ROS ↑ ERK1/2 RIP1-dependent necroptosis DNA damage	[101]
Acacetin (low doses)	MCF-7 cell in vitro	Proliferation induction (in low doses)	↑ ERK1/2 ↑ PI3K/Akt	[102]
Silymarin	MDA-MB- 231 and MCF-7 cells in vitro and MCF-7 tumors in vivo	Inhibition of proliferation and induction of apoptosis	↑ Bax, cleaved poly-ADP ribose polymerase, cleaved caspase-9, and p-JNK ↓ Bcl-2, p-P38, and p-ERK1/2	[103]
Puerarin	MCF-7 and MDA-MB-231 cell in vitro	Suppression of cell migration, invasion, and adhesion	↓ NF-κB, p65, IκBα, and pERK1/2	[104]
Sophorafla-vanone G	MDA-MB-231 cells in vitro	Apoptosis induction	↓ phosphorylation of Akt, p38, ERK1/2, and JNK ↑ cleaved caspase-8, caspase-3, and caspase-9 ↓ Bcl-2 and Bcl-xL	[105]
Glabridin	MDA-MB-231 cells in vitro	Decrease in cell migration, invasion, and angiogenesis	↓ FAK/Src complex Akt, and ERK1/2, ↓ RhoA and myosin light chain phosphorylation	[106]
Nobiletin	MCF-7, SK-BR-3, and MDA-MB-468 cells in vitro	Cell cycle arrest	↓ ERK1/2, cyclin D1, ↑ p21	[107]
Baicalein	MCF-7, and SK-BR-3 cells in vitro	Suppression of cell migration, invasion, and adhesion	↓ EGFR, ERK1/2, and Akt	[108, 109]
Fisetin	MCF-7 cells in vitro	Suppression of cell invasion	↓ NF-κB ↓ PKCα/ROS/ERK1/2 and p38 MAPK ↓ MMP-9 expression	[110]
Genistein	MDA-MB-231 cells in vitro	Cell cycle arrest	↑ ERK1/2 c-Jun and c-Fos	[111]
Isorhamnetin	MCF-7 and MDA-MB-468 cells in vitro	Apoptosis induction	↓ Akt/mTOR and MEK/ERK1/2 pathways ↑ Bax/Bcl-2 and cleaved caspase-3	[112]

↓ indicates decreased/suppressed; ↑ indicates increased/enhanced. *Akt*, Protein kinase B; *AP-1*, Activating protein-1; *b2AR*, Beta-2 adrenergic receptor; *EGCG*,: Epigallocatechin-3-gallate; *EGFR*, Epidermal Growth Factor Receptor; *ERK*, Extracellular signal-regulated kinase; *FAK/SRC*, Focal adhesion kinase and steroid receptor coactivator complex; *FOXM1*, Forkhead box M1; *GSK-3b*, Glycogen synthase kinase-3 beta; *HER2*, Human epidermal growth factor receptor 2; *IGF1R*, Insulin-like growth factor 1 receptor; *IκBα*, Inhibitor of the nuclear transcription factor *NF-κB*; IRS, Insulin receptor substrate; *JNK*,: c-Jun N-terminal kinase; *MEK1*, Mitogen-activated protein kinase 1; *MMP*, Matrix metallopeptidase; mTOR,: Mammalian target of rapamycin; *NF-κB*, Nuclear factor kappa B; *PAK1*, p21 (RAC1) activated kinase 1; *PCNA*, Proliferating cell nuclear antigen; *PI3K*, Phosphoinositide 3-kinase; *PKC*,: Protein kinase C; *PTGS2*, Prostaglandin-endoperoxide synthase 2; *p38*, Mitogen-activated protein kinase; *RhoA*, Ras homolog family member A; *RIP1*, Ribosome Inactivating Protein 1; *ROS*, Reactive oxygen species

### Flavonoids and the JNK signaling pathway

So far, data on the involvement of flavonoids in the JNK pathway in BC come almost exclusively from in vitro studies that mainly used the hormone-positive MCF-7 cell line. In 2000 Leung and Wang reported sustained activation of JNK (also referred to as stress-activated protein kinases, SAPKs) in genistein-treated MCF-7 cells (25 and 50 μmol/L), which positively correlated with apoptotic response. The authors suggested that genistein-induced apoptosis was somewhat due to modulation of the stress pathway than the pathway mediated by the Bcl-2 family [113]. On the other hand, decreased proliferation in another hormone-dependent BC cell line T47D after therapy with higher concentrations of genistein (25 μM to 100 μM) was not associated with p-JNK expression; however, a decrease in phosphorylated p90RSK (a downstream effector protein of the MAPK pathway) was found (after treatment with 100 μM) [114]. Similarly, genistein (50–100 μM) and quercetin (25–100 μM) inhibited MCF-7 vec and MCF-7 HER2 cell growth independent of AKT, ERK, JNK, and p38. The authors attributed their antiproliferative activity to NF-κB signaling inhibition [115]. It is possible that modulation of the MEK/ERK5 signaling pathway, which is discussed below, largely contributes to the oncostatic efficacy of genistein in BC. This remains to be elucidated, and the optimal dose of genistein must be determined.

Quercetin (100 μM) increased p-JNK and activated caspases in T47D cells; treatment with JNK inhibitor SP600125 partially suppressed quercetin-induced apoptosis [116]. In another study, quercetin (25 and 50 μM) and other flavonoids, baicalein (25 and 50 μM), and 3-OH flavone (25 and 50 μM) inhibited E2/IGF-1-induced proliferation of MCF-7 cells via suppressed phosphorylation of IRS-1, ERKs, and JNKs proteins [117]. A quercetin derivate isorhamnetin (20 and 40 µM) reduced the adhesion, migration, and invasive capabilities of MDA-MB-231 cells through MMPs downregulation without affecting JNK and ERK1/2, but a decrease in p-p38 and p-STAT3 was found [118].

Crude catechin containing approximately 53% of EGCG, a natural inhibitor of GRP78, inhibited the growth of T47D cells (30 and 100 μg/mL), increased p-JNK and p-p38, and modulated expression of CDK, cyclin A, and cyclin B1 proteins leading to G2 arrest [119]. The dose-dependent effect of EGCG on ROS generation and the mechanism of cell death in MCF-7 cells was reported. Cells exposed to 10–50 μM EGCG exhibited substantial ROS generation and alterations in mitochondrial membrane potential, whereas no such effects were observed in untreated controls or cells treated with 100–400 μM EGCG. Lower concentrations activated JNK and induced apoptosis, whereas higher concentrations triggered necrosis and did not affect JNK. Moreover, the authors showed that intracellular ATP levels were inversely associated with EGCG dose. These findings support the hypothesis that ATP functions as a regulatory switch in modulating EGCG-induced ROS production and the subsequent activation of apoptotic pathways [120]. Exposure of MCF-7 cells to EGCG (10 μM) potentiated taxol (paclitaxel) and vinblastine-induced increase in apoptosis via p-JNK, activation of caspase-7, and PARP cleavage. Inhibition of JNK and caspase-7 abrogated EGCG sensitization [121]. The hypothesis that EGCG may improve the effect of microtubules-interfering chemotherapeutics is supported by another study by Luo et al. where EGCG counteracted paclitaxel-induced upregulation of GRP78 expression and increased paclitaxel-induced p-JNK both in murine triple-negative 4T1 mammary cancer cells (20 μM) and BALB/c mice carrying 4T1 transplants (30 mg/kg/day *i.p*. for 24 days) [122].

Flavone (25 μM) and chalcone (25 μM), abundantly present in fruits and vegetables, inhibited the activity of ERα through the stimulation of JNK1 and JNK2 in MCF-7 cells. The anti-estrogenic effects of chalcone and flavone required intact JNK signaling as the constitutive activation of the JNK pathway suppressed E2-mediated gene expression [123].

Elevated FAS expression is associated with HER2 overexpression, which is a poor prognostic marker in BC. Amentoflavone (75 and 100 μM), a biflavonoid present in various medicinal plants, including *Ginkgo biloba* L., suppressed FAS expression and HER2 activation, inhibited the phosphorylation of AKT, mTOR, and JNK, diminished cell viability, and triggered apoptosis in TNBC SKBR3 cells [124].

Apigenin (40 μM), a non-mutagenic flavonoid with low toxicity and widespread presence in various fruits, suppressed HGF-induced invasive growth and metastasis in the MDA-MB-231 cell line. This effect occurred independently of Met, ERK, and JNK phosphorylation, with the anti-invasive properties likely resulting from the inhibition of the PI3K/Akt pathway and β4 integrin function [125]. Chen et al. compared the effect of apigenin (30 and 60 μM) with its natural derivate protoapigenone (3 and 10 μM) in the same cell line and found that exposure to both substances led to the loss of mitochondrial membrane potential but protoapigenone-induced apoptosis with tenfold greater potency. Protoapigenone, but not apigenin, induced Bcl-2 and Bcl-xL phosphorylation and activation of ERK, JNK, and p38. MAPK inhibitors prevented these effects [126]. Another derivate of apigenin, acacetin (50–200 μM), increased ROS generation and induced apoptosis in MCF-7 cells. Activation of SAPK/JNK1/2 and c-Jun but neither ERK1/2 nor p38 activation was found after acacetin treatment [127].

Kim and Jung investigated the effect of chrysin, a dihydroxyflavone found in honey, propolis, and various plant sources, in a mouse xenograft model. Chrysin nanoparticles given to BALB/c nude mice (three times a week at a dose of 10 mg/kg *i.v.* for 20 days) decreased the proliferation of MDA-MB-231 xenografts through the suppression of PI3K/JNK signaling pathway and induced apoptosis via the p53-pathways. Tumor tissues from chrysin-treated mice also showed downregulated expression of MMPs, which led to decreased metastasis. These results point to the potential use of chrysin as an adjuvant therapy in TNBC [128].

Hesperitin (160 μM), a trihydroxyflavone mostly found in citrus fruits, induced apoptosis by ROS accumulation and activation of the ASK1/JNK pathway in MCF-7 cells [129]. On the other hand, nobiletin (2–16 μM), a polymethoxy flavone abundant in citrus fruits, inhibited the cell cycle, migration, and invasiveness of MCF-7 and T47D cells. The authors attributed these effects to downregulating the IL-6-activated ERK/STAT and JNK/c-JUN pathways. Nobiletin (30 or 60 mg/kg, given every other day, *per os* for 4 weeks) also reduced BC proliferation and metastasis in BALB/c nude mice carrying MCF-7 transplants [130].

Luteolin (5–25 μM) decreased p-JNK and aromatase activity in MCF-7 cells [131]. A glycosylflavone of luteolin, mLU8C-PU (10–40 μM), inhibited the invasion of 12-O-tetradecanoylphorbol-13-acetate (TPA)-treated MCF-7 cells via decreased p-JNK, membrane translocation of PKCα, and the nuclear translocations of AP-1 and NF-κB [132]. On the other hand, luteolin 8-C-β-fucopyranoside, a C-glycosyflavone (5–20 μM), inhibited the invasion of TPA-treated MCF-7 cells without affecting JNK and p38 phosphorylation. The authors concluded that a decrease in ERK/AP-1 and ERK- NF-κB signaling was the underlying mechanism [133]. Similarly, silibinin (100 μM), a flavonolignan present in milk thistle seeds, inhibited TPA-induced ERK phosphorylation but not JNK and p38 phosphorylation and decreased metastasis via decreased MMP-9 and VEGF expression in MCF-7 cells [134].

Treatment of MCF-7 and SKBR3 cells with juglanin (2.5–10 μM), a flavonol mainly extracted from *Juglans mandshurica* L., induced G2/M phase arrest and triggered both apoptotic and autophagic pathways via enhancement of ROS/JNK signaling pathway. In the same study, juglanin (10 or 20 mg/kg/day *i.p.* given for 7 days) also decreased the growth of MCF-7 xenografts in BALB/c-nude mice. Tumour samples showed upregulated levels of cleaved caspase-9 and caspase-3, LC3BI, LC3BII, and p-JNK [135].

Icariin (12.5–50 μM), a flavonol glycoside isolated from *Epimedium brevicornum* L., reduced proliferation and invasion and activated apoptosis in Hs 578 T and MDA-MB-468 cells via ROS-mediated suppression of the JNK/c-JUN pathway [136].

Capillarisin (25–100 μM) is one of the main bioactive compounds of *Artemisia capillaris* L. [137] and delphinidin (60 μg/mL) [138] decreased phorbol 12-myristate 13-acetate (PMA)-induced MMP-9 expression, thus decreasing the invasion of MCF-7 cells; both reports showed inhibition of JNK and p38 pathways [137, 138].

Myricetin (10 and 20 μM), a flavonol in many fruits and vegetables, reduced cell viability and induced apoptosis and autophagy in the SKBR3 line via MAPK regulation. The expression levels of p-JNK and p-p38 increased, but p-ERK expression decreased after myricetin treatment [139]. Lee et al. evaluated the effect of another flavonol morin (100–200 μM) isolated from *Cudrania tricuspidata* L. and Moraceae family in the same line. Morin-induced apoptosis and suppressed cell migration and invasion. The authors attributed the reductions in cell viability to the inhibition of the HER2/EGFR signaling pathway and an increase in p-JNK and p-p38 [140]. The isoflavone scandenolone (10 and 15 μM), also found in *Cudrania tricuspidata* L. fruit, decreased the viability of MCF-7 cells, induced a mitotic cell cycle arrest, decreased mitochondrial membrane potential, and promoted apoptosis. The activation of p38 and ERK phosphorylation was found, but neither JNK nor AKT was affected. Decreased cancer cell growth and induction of apoptosis were also confirmed in vivo (5 and 7.5 mg/kg *i.v.* given every other day for 28 days) in nu/nu mice carrying MCF-7 xenografts [141].

Chen et al. investigated the effect of another isoflavone, calycosin (50 and 100 μM), in both ER-positive (MCF-7, T-47D) and ER-negative (MDA-231, MDA-435) cells. Calycosin selectively suppressed cell proliferation and induced apoptosis in ER-positive cells by activating p38 while inhibiting Akt signaling and PARP-1 cleavage, with no detectable impact on JNK activity. The authors determined that calycosin suppressed the growth of ER-positive cells through ERβ-mediated inhibition of IGF-1R, accompanied by the selective modulation of the MAPK and PI3K/Akt signaling pathways [142]. Selective MAPK regulation was also reported in MCF-7 cells treated with rotenone, an insecticidal isoflavone found in the *Derris* and *Lonchocarpus* species. Rotenone (5 µM) suppressed proliferation and induced ROS-mediated apoptosis associated with JNK and p38 activation and ERK1/2 inactivation. Suppression of JNK and p38 protected BC cells against rotenone-induced apoptosis [143].

Cyanidin-3-glucoside (40 μM) or kuromanine, an anthocyanin mainly found in black rice, inhibited ethanol-induced p-JNK and p130Cas/JNK association, which attenuated migration/invasion of MCF-7 cells [144]. Thus, cyanidin-3-glucoside may be considered as a preventive agent in ethanol-induced BC metastasis.

Apart from licochalcone D (further mentioned in chapter 3.4), another chalcone derivate, 2-hydroxychalcone (30 μM), enhanced autophagy and initiated apoptosis in MCF-7 and CMT-1211 canine mammary cancer cells. The authors found excessive intracellular ROS accumulation, induction of endoplasmic reticulum stress, and triggering of the JNK pathway. The oncostatic effect was also observed in vivo in BALB/c mice carrying CMT-1211 xenografts. 2-hydroxy chalcone (20, 40, and 60 mg/kg *i.p*. every other day for 24 days) inhibited tumor growth and metastasis and induced apoptosis through several mechanisms, including upregulated p-JNK [145].

To summarise, preclinical studies do not univocally establish the involvement of JNK signaling in BC growth suppression by flavonoids, as controversial results have been reported even when the same flavonoid was used in the same cell line. In addition*, *in vivo data are scarce. As the in vivo environment is much more complex and variable, further experiments are warranted to elucidate the impact of flavonoids on JNK and other MAPK pathways and justify their value in BC management. Table 2 highlights the effects of flavonoids on BC via MAPK-related signaling pathways.

**Table 2:** Summary of the effects of flavonoids on BC through modulation of JNK Upstream and downstream signaling pathways

Flavonoid	Study details	Affected process	Mechanism	Ref
Genistein	MCF-7	↑ apoptosis	↑ JNK	[113]
Genistein	T47D	↓ cell proliferation	↓ p-p90RSK (MAPK independent)	[114]
Genistein, quercetin	MCF-7 vec MCF-7 HER-2	↑ extrinsic apoptosis (AKT/MAPK independent)	↓ NF-κB signaling	[115]
Quercetin	T47D	↑ apoptosis	↓ HSP70 ↑ GRP78 ↑ p-JNK ↑ caspases	[116]
Quercetin, baicalein, 3-OH flavone	MCF-7	↓ E2/IGF-1-induced proliferation	↓ p-IRS-1 ↓ p- ERK ↓ p-JNK	[117]
Isorhamnetin	MDA-MB-231	↓ adhesion, migration, and invasion	↓ MMPs ↓ p-p38 ↓ p-STAT3 no effect on JNK and ERK1,2	[118]
EGCG	T47D	G2 arrest	↑ p-JNK ↑ p-p38	[119]
EGCG	MCF-7	10–50 μM: ↑ apoptosis 100–400 μM: ↑ necrosis	↑ ROS generation ↑ JNK no effect on ROS generation no effect on JNK	[120]
EGCG	MCF-7	↑ paclitaxel and vinblastine-induced apoptosis	↑ p-JNK ↑ caspase-7 ↑ PARP cleavage	[121]
EGCG	murine 4T1 cells, in vitro and in vivo (BALB/c nude mice)	↑ paclitaxel-induced apoptosis	↓ GRP78 ↑ p-JNK	[122]
Flavone, chalcone	MCF-7	↓ ERα activity	↑ JNK	[123]
Amentoflavone	SKBR3	↓ cell viability ↑ apoptosis	↓ FAS expression ↓ HER-2 activation ↓ p-AKT ↓ p-mTOR ↓ p-JNK	[124]
Apigenin	MDA-MB-231	↓ HGF-promoted growth and metastasis	↓ PI3/Akt pathway ↓ β4 integrin function no effect on p-Met, p-ERK and p-JNK	[125]
Apigenin	MDA-MB-231	↑ apoptosis	loss of mitochondrial membrane potential no effect on the expression of Bcl-2 family proteins	[126]
Protoapigenone	MDA-MB-231	↑ apoptosis	loss of mitochondrial membrane potential ↑ Bcl-2 family proteins ↑ ERK, JNK, and p38	[126]
Acacetin	MCF-7	↑ apoptosis	↑ ROS generation ↑ JNK no effect on ERK1/2 and p38	[127]
Chrysin	MDA-MB-231 xenografts in Balb/c nude mice	↓ tumor growth ↑ apoptosis ↓ metastasis	↑ p53 ↓ PI3K/JNK	[128]
Hesperetin	MCF-7	↑ apoptosis	↑ ROS generation ↑ ASK1/JNK	[129]
Nobiletin	MCF-7 T47D MCF-7 transplants in Balb/c nude mice	↓ proliferation, migration, and invasion ↓ proliferation and metastasis	↓ of IL-6-induced ERK/STAT and JNK/cJun ↓ Ki-67 ↓ p-PI3K ↓ p-ERK ↓ p-STAT3	[130]
Luteolin	MCF-7	↓ aromatase activity	↓ CYP19 expression ↓ p-JNK	[131]
mLU8C-PU	TPA-treated MCF-7 cells	↓ invasion	↓ p-JNK ↓ membrane translocation of PKCα ↓ nuclear translocations of AP-1 and NF-κB	[132]
Luteolin 8-C-β-fucopyranoside	TPA-treated MCF-7 cells	↓ invasion	no effect on p-JNK and p-p38 ↓ ERK/AP-1 ↓ ERK- NF-κB	[133]
Silibinin	TPA-treated MCF-7 cells	↓ metastasis	↓ MMP-9 ↓ VEGF expression ↓ p-ERK no effect on p-JNK and p-p38	[134]
Juglanin	MCF-7 SKBR3 MCF-7 xenografts in BALB/c nude mice	G2 arrest ↑ apoptosis ↑ autophagy ↓ tumour growth ↑ apoptosis	↑ ROS generation ↑ JNK ↑ p-JNK ↑ LC3BI and LC3BII	[135]
Icariin	Hs 578 T MDA-MB-468	↑ apoptosis ↓ invasion	↑ ROS generation ↓ JNK/c-JUN	[136]
Capillarisin	PMA-treated MCF-7 cells	↓ invasion	↓ MMP-9 ↓ p-JNK and p38	[137]
Delphinidin	PMA-treated MCF-7 cells	↓ invasion	↓ MMP-9 ↓ p-JNK and p38	[138]
Myricetin	SKBR3	↑ apoptosis ↑ autophagy	↑ p-JNK and p-p38 ↓ p-ERK	[139]
Morin	SKBR3	↑ apoptosis ↓ invasion	↓ HER2/EGFR ↑ p-JNK and p-p38	[140]
Scandenolone	MCF-7 MCF-7 xenografts in nu/nu mice	mitotic cell cycle arrest ↑ apoptosis ↑ apoptosis	↑ p38 and ERK no effect on JNK and AKT ↓ pro-caspase 3	[141]
Calycosin	MCF-7, T47D MDA-231, MDA-435	↓ proliferation ↑ apoptosis no effect	↓ IGF-1R ↑ p38 ↓ Akt ↓ PARP-1 cleavage no effect on JNK	[142]
Rotenone	MCF-7	↑ apoptosis	↑ ROS generation ↑ JNK and p38 ↓ ERK1,2	[143]
Cyanidin-3-glucoside	ethanol-treated MCF-7 cells	↓ migration/invasion	↓ p-JNK ↓ p130Cas/JNK association	[144]
2-hydroxychalcone	MCF-7 canine CMT-1211 CMT-1211 xenografts in Balb/c mice	↑ autophagy ↑ apoptosis ↓ tumour growth ↓ metastasis ↑ autophagy ↑ apoptosis	↑ ROS generation ↑ endoplasmic reticulum stress ↑ JNK ↓ p-IκB and p-NF-κBp65 ↓ MMP-9 ↑ LC-3II ↑ cleaved PARP ↑ p-JNK	[145]

↓ indicates decreased/suppressed/induction; ↑ indicates increased/enhanced; p- indicates a phosphorylated form. *Akt*, protein kinase B; *AP-1*, activator protein-1; *CYP19*, cytochrome P450 family 19; *E2*, estradiol; *EGCG*, epigallocatechin-3-gallate; *ERα*, estrogen receptor α; *ERK*, extracellular signalregulated kinase; *FAS*, fatty acid synthase;* GRP78*, glucose-regulated protein 78; *HER2*, human epidermal growth factor receptor 2; *HGF*, hepatocyte growth factor; *HSP70*, heat shock protein 70; *IGF1*,: insulin-like growth factor 1; *IL-6*, interleukin-6; *IRS-1*, insulin receptor substrate 1; *IκBα*, inhibitor of the nuclear transcription factor NF-κB; *IRS-1*, insulin receptor substrate 1; *JNK*, c-Jun Nterminal kinase; *LC-3II*, LC3-phosphatidylethanolamine conjugate; *MAPK*, mitogen-activated protein kinase; *mLU8C-PU*, 7-methoxy-luteolin-8-C-β−6-deoxy-xylo-pyranos-3-uloside; *MMP*, matrix metalloproteinase; *mTOR*, mammalian target of rapamycin; *NF-κB*, nuclear factor-kappa B; P130Cas, Crk-associated substrate protein; p38, p38 mitogen-activated protein kinases; PARP, poly (ADPribose)polymerase; *PI3K*, phosphatidylinositol 3-kinase; *ROS*, reactive oxygen species; *STAT*, signal transducer and activator of transcription; *TPA*, 12-O-tetradecanoylphorbol-13-acetate; *VEGF*, vascular endothelial growth factor

### Flavonoids and the p38 signaling pathway

A study by Liu et al. summarized nobiletin’s antiproliferative and pro-apoptotic properties in BC. This flavone glycoside-induced apoptosis by regulating apoptotic proteins (Bcl-2, Bax, caspase-3) and activating the p38 signaling pathway. The findings revealed that nobiletin activated p38 MAPK in the MCF-7 BC cell line, suppressing cell migration and promoting apoptotic processes [146]. Ginkgetin, a biflavonoid derived from *Ginkgo biloba* L., exhibits potent anti-inflammatory, antioxidant, and anticancer characteristics. Cao et al. investigated the antitumor mechanisms of ginkgetin in three human BC cell lines (MCF-7, MDA-MB-231, and BT-474). Their findings demonstrated that ginkgetin upregulated the expression of phosphorylated p38 (p-p38), c-Jun N-terminal kinase (p-JNK), and extracellular signal-regulated kinase (p-ERK) in vitro. Additionally, more p-p38-, p-ERK, and p-JNK-positive cells were detected in tumor tissues in vivo (MCF-7 xenograft mice)*.* These findings indicate that the anticancer effects of ginkgetin in BC are largely mediated through the activation of MAPK signaling pathways [147].

Flavonoids such as baicalin and baicalein have been described to induce apoptosis and suppress BC cell migration through the p38 MAPK signaling pathway. Notably, their combined treatment exerted a synergistic effect, significantly enhancing apoptosis in the MCF-7 cell line. These compounds’ pro-apoptotic and antiproliferative activities were attributed to the modulation of p38 and ERK proteins. Furthermore, the inhibitory effect on cell growth was markedly reduced upon treatment with specific inhibitors of p38 MAPK (SB203580) and ERK (PD98059) [148]. Additionally, several studies have confirmed the independent antitumor activity of baicalin in BC via the p38 MAPK pathway [149, 150]. Wang et al. summarized that baicalin effectively suppressed migration, invasion, and metastasis in MDA-MB-231 BC cells and the xenograft model [150]. Moreover, a recent study investigated the anticancer potential of baicalin in the human medullary BC cell line Bcap-37, highlighting its role in modulating the ERK/p38 signaling pathway [149].

The bioactive compound wogonin has been reported to show anticancer, antiviral, anti-inflammatory, and neuroprotective effects. Yu and Kim demonstrated that wogonin treatment increased reactive oxygen species (ROS) generation in the MCF-7 BC cell line, which is linked with MAPK upregulation. Notably, the activation of ERK and p38 MAPK induced by wogonin was suppressed by N-acetylcysteine (NAC), a ROS scavenger, indicating that wogonin-induced ROS generation plays a key role in MAPK pathway activation [151]. Wogonoside, a glucuronide derivative of wogonin, has also been identified as a potential anticancer agent. Sun et al. investigated its anti-angiogenic properties in the MDA-MB-231 BC cell line, revealing that these effects were linked to increased expression of LC3II and Beclin-1, markers indicative of autophagy activation. Furthermore, wogonoside modulated the expression of p-ERK and p-p38, leading to mTOR inhibition and subsequent activation of autophagy [152]. Eriocitrin, a flavanone derived from lemons, has been documented to have pro-apoptotic and antiproliferative effects in MCF-7 BC cells via modulation of the STAT3/MAPK signaling. As previously noted, JNK/p38 activation can influence oxidative stress within cells. Similarly, eriocitrin enhanced ROS generation via protein kinase-mediated mechanisms, ultimately inducing apoptotic cell death in MCF-7 cells [153].

Another study focused on the activity of tyrosine kinases as crucial regulators of apoptotic and metastatic signaling pathways in BC cells. Proteome profiling of MDA-MB231 cells revealed an upregulation of p38α phosphorylation in response to flavokawain B treatment. This chalcone is noteworthy due to its diverse biological activities, including anti-nociceptive, anti-inflammatory, and promising anticancer properties. The study demonstrated that Flavokawain B induced G2/M phase cell cycle arrest, promoted apoptosis, and suppressed metastasis [154]. Licochalcone D, another chalcone derivative, exhibited cytotoxic effects on BC cells. Furthermore, this natural compound increased the sensitivity of BC cells to tumor necrosis factor-related apoptosis-inducing ligand (TRAIL) by upregulating death receptor 5 (DR5). Notably, the increased DR5 expression was mediated by JNK/p38 MAPK activation [155]. Similarly, butein, another chalcone derivative, was shown to suppress MDA-MB-231 cell proliferation through ROS generation and the regulation of p38 and ERK signaling pathways [156]. These findings (Table 3) highlight the p38 MAPK pathway as a promising therapeutic signaling target for developing novel natural compounds with potential anticancer and chemosensitizing effects against resistant BC.

**Table 3:** Flavonoids’ impact on BC cells specifically via regulation of MAPK-p38 and -ERK5 signaling pathways

Flavonoid	Study details	Affected process	Mechanism	Ref
Nobiletin	MCF-7 cells in vitro	Suppression of cell migration and promotion of apoptosis	↓ Bcl-2 ↑ Bax, p53, cleaved caspase-3 ↑ p38 phosphorylation	[146]
Ginkgetin	MCF-7, MDA-MB-231, and BT-474 cells in vitro, MCF-7 xenograft mice	Growth inhibition and apoptosis induction of BC	↑ p38 phosphorylation ↑ JNK phosphorylation ↑ ERK phosphorylation	[147]
Baicalin and Baicalein	MCF-7 cells in vitro	Apoptosis induction and inhibition of BC cell migration	↑ p38 phosphorylation ↑ ERK phosphorylation	[148]
Baicalin	B-cap-37 cells in vitro	Inhibition of proliferation and migration of BC	↑ p38 expression ↑ p-ERK1 expression ↑ Bax expression ↓ Bcl-2 expression	[149]
Baicalin	MDA-MB-231 cells in vitro and xenograft model	Suppression of migration, invasion, and metastasis of triple-negative BC	↓ p38	[150]
Wogonin	MCF-7 cells in vitro	Apoptosis of BC	↑ ROS generation ↑ p38 and ERK activation	[151]
Wogonoside	MDA-MB-231 cells in vitro	Suppression of angiogenesis and autophagy induction	↑ expression of LC3II, Beclin-1 ↑ p38 and ERK phosphorylation ↓ mTOR	[152]
Eriocitrin	MCF-7 cells in vitro	Induction of apoptotic cell death and inhibition of proliferation	↑ ROS generation ↑ p38 and JNK activation	[153]
Flavokawain B	MDA-MB-453 cells in vitro	Induction of cell cycle arrest and apoptosis, metastasis inhibition	↑ p38α expression G2/M phase cell cycle arrest	[154]
Licochalcone D	BC cells in vitro	Induction of apoptosis	↑ DR5 expression ↑ p38 and JNK activation	[155]
Butein	MDA-MB-231 cells in vitro	Inhibition of proliferation	↑ ROS generation ↑ p38 and ERK signaling	[156]
Genistein	MDA-MB-231 cells in vitro	Activation of apoptosis and inhibition of proliferation	↓MEK5, ERK5 and pERK5 ↓NF-κB/p65 protein levels	[158]
Genistein	MCF-7 cells in vitro	Suppression of carcinogenesis	↓MEK/ERK5 signaling	[159]
Kaempferol, Quercetin, and ( +)-Catechin	BC – specific combinational QSAR model	Inhibition of BC	↓ ERK5	[160]

↓ indicates decreased/suppressed; ↑ indicates increased/enhanced. *Bax*, Bcl-2-like protein 4; *Beclin-1*, Bcl-2 interacting protein; *Bcl-2*, B-cell lymphoma-2; *DR5*, death receptor 5; *ERK*, Extracellular signal-regulated kinase; *JNK*, c-Jun N-terminal kinase; *QSAR*, Quantitative Structure-Activity Relationship; *LC3II*, Microtubule-associated protein 1A/1Blight chain 3; *MEK*, Mitogen-activated protein kinase; *mTOR*, Mammalian target of rapamycin; *NF- κB*, Nuclear factor kappa B; p38, Mitogen-activated protein kinase; *p53*, Tumor protein p53; *ROS*, Reactive oxygen species

### Flavonoids and the ERK5 signaling pathway

The flavonoid genistein has been extensively evaluated for its regulatory effects on cell proliferation and apoptosis [157]. In MDA-MB-231 cells, genistein downregulated the protein levels of MEK5, total ERK5, and phosphorylated ERK5 (pERK5) concentration-dependently. Furthermore, it reduced NF-κB/p65 protein levels and suppressed the DNA-binding activity of NF-κB, indicating that the suppression of the MEK5/ERK5/NF-κB signaling pathway contributes to its pro-apoptotic effects [158]. Additionally, a novel network-based analytical approach for ranking plant polyphenols has identified genistein as a potent modulator of the MEK5/ERK5 pathway in MCF-7 BC cell lines [159].

Recent in silico studies have identified flavonoids derived from *Blighia sapida* L. as potential therapeutic agents targeting ERK5, a critical regulator of BC progression. These compounds were discovered through molecular docking, molecular mechanics with generalized Born and surface area solvation, and pharmacokinetic modeling. Their inhibitory potential was further evaluated using density functional theory and *pIC50* value analysis. Bodun et al. demonstrated that kaempferol, quercetin, and ( +)-catechin exhibited the highest docking scores, suggesting their strong potential as ERK5 inhibitors for BC therapy [160].

Given the established role of ERK5 in BC pathophysiology (Table 3), flavonoids emerge as promising therapeutic candidates in the ongoing search for effective treatment strategies in resistant BC.

## Clinically relevant illustration in the framework of 3P medicine

This subchapter provides clinically relevant examples to illustrate the association of BC development and progression with MAPK signaling pathways in the framework of 3PM.

### Case reports

#### Case report 1 – MAPK-relevant disease predisposition: AI-prediction based on systemic effects detected by multi-level diagnostics

A 26-year-old premenopausal female patient demonstrated benign (BC-free) breast alterations. The patient underwent a non-invasive comprehensive blood test linked to stratification algorithms differentiating between low and high risk of BC predisposition. The AI-based algorithm dedicated to the preBC risk assessment was developed specifically for the premenopausal patient cohort diagnosed with BC-free breast benignancy [161]. The blood test revealed high-risk characteristically increased levels of Catalase and Actin (proteomics), as well as the ratio Hcy to the Comet Assay (CA), patterns CA-I/CA-IV (hybridome), whereas the CA-IV, CA-III, CA-II patterns as well as the ratio CA-I/CA-IV, were high-risk characteristically decreased. Consequently, the patient was stratified as being at high risk of BC development. Intensive protective measures with regular monitoring were recommended. Relevance to the MAPK-signalling: stress sensitivity, pro- vs. anti-apoptotic regulation – all directly related to the MAPK-signalling [162–164]. Consequently, stress-relevant MAPK signaling pathways represent an attractive target for therapeutic intervention in vulnerable individuals (primary care).

### Case report 2 – MAPK-relevant patient phenotyping in primary and secondary care

A female postmenopausal patient, 58 years old with BMI = 20.8, was diagnosed with metastatic BC: T3N1M1. Despite the chemotherapeutic treatments applied, an aggressive metastatic disease affected the liver, further expanding to the bones and lungs. The patient is an evident Flammer Syndrome Phenotype (FSP) carrier demonstrating the following characteristic patterns:

**Table:** 

Cold hands and/or feet	Yes	frequently
Feel cold	Yes	frequently
Low blood pressure	Yes	
Dizziness	Yes	e.g. by standing up
Prolonged sleep onset	Yes	shifted circadian rhythms
Migraine with aura	Yes	strongly pronounced
Altered reaction towards drugs	Yes	strongly pronounced
Pronounced pain sensitivity	Yes	
Strong smell perception	Yes	strongly pronounced
Slim at 20–30 years of age	Yes	
Specific psychosomatic patterns	Yes	strongly pronounced meticulous personality
Tinnitus	Yes	
Reversible skin blotches in stress	Yes	strongly pronounced
Impaired wound healing	Yes	strongly pronounced
Reciprocity between FSP and MAPK-signalling in the context of aggressive BC with potential drug resistance: - Thermal regulation [165–167] - Shifted circadian rhythms [168–172] - Disturbed microcirculation linked to systemic ischemia-reperfusion [170, 173–176] - Specific psychosomatic patterns with strongly pronounced perfectionism [168, 173, 177] - Stress vulnerability [165, 168, 177, 178] - Migraine [168, 170, 179] - Altered drug sensitivity & shifted MDR transporter profiles [4, 168, 170, 180–182] - Impaired wound healing [173, 183–187]		

### Case report 3 – MAPK-relevant metastatic BC treatment monitoring

A female patient, 39 years of age, diagnosed with triple-negative BC and metastatic disease in the liver, was treated with SIRT. The patient died within 3 months after the treatment was applied.

The patient underwent blood tests before the treatment, which revealed.

- significantly increased activity rates of both MMP-9 and MMP-2 in blood plasma
- low level of the Comet Assay class CA-I in circulating leukocytes
- high level of the Comet Assay class CA-III in circulating leukocytes
- high level of the Comet Assay class CA-IV in circulating leukocytes
- low level of SOD-2 in circulating leukocytes
- low level of Catalase in circulating leukocytes
- high level of Calgranulin A in circulating leukocytes
- low level of Profilin in circulating leukocytes
- low level of RhoA in circulating leukocytes.

Her molecular patterns corresponded to an extremely poor prognosis according to the multi-parametric risk assessment elaborated for this patient cohort [188, 189]. Relevance to the MAPK-signalling is reflected in this disease-specific molecular panel including stress- and apoptosis-relevant indicators [4, 190], metalloproteinase activity patterns [191, 192], regulation of SOD-2 [193, 194], Catalase [194, 195], Calgranulin A [196–198], Profilin [199, 200], and RhoA [201–203].

In general, patients with hepatic BC metastases were demonstrated to have disease-specific increases in the activity rates of both MMP-9 and MMP-2 and highly characteristic molecular patterns under the SIRT treatment when compared to all other stratified sub-groups with liver metastases [189]. This discovery makes metastatic BC treatment particularly challenging and requires tailored treatment approaches considering the key pathways involved.

### Concluding remarks with clinically relevant outlook

Plant-derived natural compounds exhibit remarkable biological activities and effectively regulate numerous signaling pathways disrupted in cancer. Preclinical studies in this domain highlight the potential of flavonoids as multifunctional agents capable of downregulating key factors involved in developing multidrug resistance (MDR). Furthermore, these compounds significantly enhance cancer cell sensitivity to conventional chemotherapeutic agents [204, 205]. The rising incidence of cancer-related morbidity and mortality has intensified the focus on improving drug sensitivity and overcoming chemoresistance in cancer cures. Traditional Chinese Medicine (TCM) has demonstrated potential as an adjunct to conventional anticancer therapies by mitigating side effects and improving therapeutic efficacy. TCM achieves this through mechanisms such as increasing intracellular accumulation of anticancer agents, inhibiting the onset of drug tolerance, modulating cell death and associated signaling pathways, improving the TME, alleviating immunosuppression, reversing epigenetic alterations, and delivering significant tumor-suppressive effects [206]. TCM and flavonoid-rich plant-based dietary interventions represent promising alternative and complementary approaches for BC management. These strategies are particularly valuable for introducing novel sensitizers to improve the effectiveness of existing anticancer therapies [207]. For instance, Yanghe Decoction, a TCM formulation, is recognized for its substantial therapeutic effectiveness in treating BC [208, 209]. Although numerous studies have suggested that natural flavonoids may serve as promising candidates for cancer therapy, only a few clinical trials have been conducted to assess their clinical benefits. In this regard, BC patients undergoing radiation therapy were administered 400 mg EGCG tablets three times daily. The impact of EGCG on cell growth, invasion, and angiogenesis was assessed by collecting blood samples at various intervals. A comparison between patients receiving radiation alone and those treated with radiotherapy and EGCG over an extended period (ranging from two to eight weeks) revealed significantly decreased blood levels of HGF, VEGF, and the activation of MMP9/MMP2 in the latter group [210]. In addition, for the treatment of other cancer types, two cycles of quercetin (420 mg/m^2^) resulted in a reduction of CA 125 levels from 295 to 55 units/mL in an ovarian cancer patient with cisplatin resistance. In contrast, serum alpha-fetoprotein levels decreased in a hepatoma patient. Furthermore, the intravenous bolus administration of quercetin at the specified dose was safe. Inhibition of lymphocyte tyrosine kinase activity and signs of anticancer effects were exhibited at the plasma concentrations achieved [211]. No clinical trials have evaluated the impact of pure flavonoids or flavonoid-enriched formulations on BC chemosensitization through the modulation of MAPK signaling pathways. A thorough clinical assessment of the anticancer mechanisms of pure flavonoids or flavonoid-rich fruits, vegetables, herbs, or spices, particularly those mediated through MAPK, is crucial for identifying potential novel adjuvant agents. These compounds have the potential to enhance BC treatment by overcoming MDR when administered in conjunction with conventional chemotherapeutic agents [212].

This article emphasizes the well-documented capacity of flavonoids to modulate MAPK signaling, a pathway intricately involved in regulating cancer cell plasticity and subsequent therapy resistance in BC (Fig. 1). Based on comprehensive preclinical evidence, we propose flavonoids as one of the most promising plant-derived compounds for targeting MAPK-driven cancer cell plasticity, a process linked to the disruption of key cellular signaling pathways that contribute to BC resistance. The flavonoid-mediated modulation of the MAPK pathway, along with its associated upstream and downstream signaling cascades, holds considerable clinical potential for regulating pro-inflammatory gene expression, plasticity of cancer cells, and chemoresistance development. These mechanisms are essential for preventing cancer cells from escaping immune surveillance, as well as inhibiting metastatic progression and recurrence. This insight paves the way for the development of innovative therapeutic strategies, including combination drug therapies aimed at effectively suppressing cancer cell plasticity [7, 21].

Integrating conventional oncology therapies using flavonoids as agents for modulating cell plasticity and enhancing chemosensitivity represents a promising strategy for addressing therapeutic resistance in BC [213]. This approach requires further investigation and refinement within preventive, predictive, and personalized oncology frameworks to advance toward complete BC management [214]. Nevertheless, preclinical studies investigating the impact of flavonoids on BC cell plasticity via MAPK signaling modulation have revealed several significant limitations. The clinical application of flavonoids as adjunct anticancer agents is obstructed by issues such as poor bioavailability, high costs and complexity of extraction processes, and obstacles in epidemiological studies, including inconsistencies in practical usage [83, 84, 215]. Additionally, flavonoids’ targeted modulation of intestinal microbiota and phase II metabolism can influence the toxicity and metabolism of other drugs, vitamins, and minerals, potentially impacting patient health. Addressing key issues is essential for advancing BC research, including (a) establishing pharmacokinetic profiles to enable effective and safe dosing, (b) developing advanced delivery systems such as nanoparticles and nano-emulsions to improve targeting and safety, (c) identifying BC subtypes sensitive to flavonoid-based interventions using a multi-omics approach tailored to individual patient characteristics, and (d) determining optimal combinations with conventional therapies to resensitize cancer cells [7, 216–218].

3PM novelty makes good use of patient phenotyping and stratification, predictive multi-level diagnostics, and application of AI to individualized interpretation of big data — all proposed for cost-effective treatments tailored to individualized patient profiles with clear benefits to patients and advanced BC management.

## Data Availability

No datasets were generated or analysed during the current study.

## Abbreviations

AI: Artificial intelligence
Akt: Protein kinase B
AP-1: Activating protein-1
b2AR: Beta-2 adrenergic receptor
Bax: Bcl-2-like protein 4
Bcl-2: B-cell lymphoma-2
Beclin-1: Bcl-2 interacting protein
CA: Comet Assay
CYP19: Cytochrome P450 family 19
DR5: Death receptor 5
E2: Estradiol
EGCG: Epigallocatechin-3-gallate
EGFR: Epidermal growth factor receptor
ERK: Extracellular signal-regulated kinase
ERα: Estrogen receptor α
FAK/SRC: Focal adhesion kinase and steroid receptor coactivator complex
FAS: Fatty acid synthase
FOXM1: Forkhead box M1
FSP: Flammer Syndrome Phenotype
GRP78: Glucose-regulated protein 78
GSK-3b: Glycogen synthase kinase-3 beta
HER2: Human epidermal growth factor receptor 2
HGF: Hepatocyte growth factor
HSP70: Heat shock protein 70
IGF1: Insulin-like growth factor 1
IGF1R: Insulin-like growth factor 1 receptor
IL-6: Interleukin-6
IRS: Insulin receptor substrate
IκBα: Inhibitor of the nuclear transcription factor NF-κB
JNK: C-Jun N-terminal kinase
LC3II: Microtubule-associated protein 1A/1B-light chain 3
MAPK: Mitogen-activated protein kinase
MEK: Mitogen-activated protein kinase
mLU8C-PU: 7-Methoxy-luteolin-8-C-β-6-deoxy-xylo-pyranos-3-uloside
MMP: Matrix metalloproteinase
mTOR: Mammalian target of rapamycin
NF-κB: Nuclear factor kappa B
P130Cas: Crk-associated substrate protein
p38: Mitogen-activated protein kinase
p53: Tumor protein p53
PAK1: P21 (RAC1) activated kinase 1
PARP: Poly (ADP-ribose) polymerase
PCNA: Proliferating cell nuclear antigen
PI3K: Phosphoinositide 3-kinase
PKC: Protein kinase C
PTGS2: Prostaglandin-endoperoxide synthase 2
QSAR: Quantitative Structure–Activity Relationship
RhoA: Ras homolog family member A
RIP1: Ribosome Inactivating Protein 1
ROS: Reactive oxygen species
SOD-2: Superoxide-dismutase 2
STAT: Signal transducer and activator of transcription
TME: Tumor microenvironment
TPA: 12-O-tetradecanoylphorbol-13-acetate
VEGF: Vascular endothelial growth factor
